# Differential Expression of Ecdysone Receptor Leads to Variation in Phenotypic Plasticity across Serial Homologs

**DOI:** 10.1371/journal.pgen.1005529

**Published:** 2015-09-25

**Authors:** Antónia Monteiro, Xiaoling Tong, Ashley Bear, Seng Fatt Liew, Shivam Bhardwaj, Bethany R. Wasik, April Dinwiddie, Carole Bastianelli, Wei Fun Cheong, Markus R. Wenk, Hui Cao, Kathleen L. Prudic

**Affiliations:** 1 Department of Ecology and Evolutionary Biology, Yale University, New Haven, Connecticut, United States of America; 2 Department of Biological Sciences, National University of Singapore, Singapore; 3 Yale-NUS College, Singapore; 4 Department of Applied Physics, Yale University, New Haven, Connecticut, United States of America; 5 Department of Biochemistry, National University of Singapore, Singapore; 6 Department of Zoology, Oregon State University, Corvallis, Oregon, United States of America; University of California Davis, UNITED STATES

## Abstract

Bodies are often made of repeated units, or serial homologs, that develop using the same core gene regulatory network. Local inputs and modifications to this network allow serial homologs to evolve different morphologies, but currently we do not understand which modifications allow these repeated traits to evolve different levels of phenotypic plasticity. Here we describe variation in phenotypic plasticity across serial homologous eyespots of the butterfly *Bicyclus anynana*, hypothesized to be under selection for similar or different functions in the wet and dry seasonal forms. Specifically, we document the presence of eyespot size and scale brightness plasticity in hindwing eyespots hypothesized to vary in function across seasons, and reduced size plasticity and absence of brightness plasticity in forewing eyespots hypothesized to have the same function across seasons. By exploring the molecular and physiological causes of this variation in plasticity across fore and hindwing serial homologs we discover that: 1) temperature experienced during the wandering stages of larval development alters titers of an ecdysteroid hormone, 20-hydroxyecdysone (20E), in the hemolymph of wet and dry seasonal forms at that stage; 2) the 20E receptor (EcR) is differentially expressed in the forewing and hindwing eyespot centers of both seasonal forms during this critical developmental stage; and 3) manipulations of EcR signaling disproportionately affected hindwing eyespots relative to forewing eyespots. We propose that differential EcR expression across forewing and hindwing eyespots at a critical stage of development explains the variation in levels of phenotypic plasticity across these serial homologues. This finding provides a novel signaling pathway, 20E, and a novel molecular candidate, EcR, for the regulation of levels of phenotypic plasticity across body parts or serial homologs.

## Author Summary

One of the most exquisite types of organismal adaptations in nature occurs when organisms are able to change the way they develop in anticipation of the different selective environments they will experience as adults. This leads to variation in adult morphologies that are adaptive. Environmental cues experienced during development often lead to variation in hormonal titers that can have profound effect on the way genes are regulated and on the adult morphology. Here we examine the hormonal and molecular mechanisms that allow specific traits that are repeated in an organism (butterfly eyespots) to either be sensitive to environmental cues–and develop different morphologies—or insensitive to these cues and develop the same morphology. We discover that a specific gene, a hormone receptor, that is expressed in the sensitive eyespots but absent in the insensitive eyespots, is responsible for regulating the level of sensitivity of each of the eyespots to an environmental cue. We identify a molecule that is regulating levels of environmental sensitivity, or phenotypic plasticity, across repeated traits in an organism.

## Introduction

Many organisms exhibit repeated traits along their bodies, or serial homologues, that look alike because they develop using a common gene regulatory network. Examples include limbs and segments in arthropods, insect’s fore and hindwings, teeth, and vertebrae. Serial homologs, however, can evolve distinct characteristics when selection favors different functions for different homologs [1]. This differentiation is achieved via local modifications to the core gene network shared across all serial homologs. Well-studied examples of this phenomenon include the modification of metathoracic dipteran wings into balancing organs [2] and the transformation of trunk walking appendages into feeding appendages in several crustaceans [3, 4], both achieved via the actions of locally expressed hox genes.

In the examples above, differentiation of serial homologues along the body axis is genetically fixed, and is independent of environmental change, e.g., the dipterans halteres will always function as a balancing organ and will always look different from the flight wings, irrespective of rearing environment. In other species, however, such as those that live in seasonal environments, serial homologous traits may be selected for similar or for different functions at different times of the year. Alternating selection pressures on the same serial homolog should lead to the evolution of phenotypic plasticity whereas constant selection should lead to fixed phenotypes [5]. Little is known, however, about the mechanisms that allow subsets of serial homologs to vary in their degree of phenotypic plasticity across a body axis.

The eyespot wing patterns on nymphalid butterflies are good candidates to investigate mechanistic questions about variation in levels of trait plasticity across serial homologs. Eyespots originally evolved as a few units on the ventral hindwings, but were later co-opted into the forewing and dorsal wing surfaces [6–8]. Due to their place of origin, the initial role of eyespots was likely in predator deflection [9], but as eyespots evolved new positions on wing surfaces they undertook novel ecological functions [7, 10–13]. Furthermore, in species that colonized seasonal environments, the same eyespots evolved phenotypic plasticity to undertake different functions at different times of the year. For example, one of the best-characterized examples of phenotypic plasticity involves changes in the size of eyespots on ventral wing surfaces of dry season (DS) and wet season (WS) forms of tropical butterflies in response to rearing temperature [14, 15]. Mark-recapture experiments in one species, *Bicyclus safitza*, showed that these plastic changes in eyespot size are adaptive [16], especially the reduction of eyespot size in the DS. The current hypothesis holds that large eyespots serve a predator-deflective function in the WS cohort, when butterflies are actively mating and laying eggs and are very visible to predators, and serve a different, cryptic, function in the DS cohort, when butterflies are primarily hiding in the dry vegetation waiting for the rainy WS to lay their eggs [15–17]. Eyespots on different wing surfaces, however, are not all equally visible to predators, and, thus, are not necessarily under the same type of alternating selection pressure. Large eyespots on the forewing ventral surfaces, for instance, are often hidden by the hindwings but can be conditionally displayed–for instance, immediately upon alighting from flight or when butterflies sense danger (S1 Fig). These eyespots potentially serve similar signaling functions–deflection of predator attacks to the wing margin—across both WS and DS environments, and selection may have favored a fixed “conspicuous” phenotype for these eyespots that is independent of rearing temperature. This fixed function would contrast with the alternating functions of eyespots that are always displayed across WS and DS environments. Forewing and hindwing eyespots at homologous positions on ventral wing surfaces may, thus, have evolved different levels of phenotypic plasticity.

**Fig 1:**
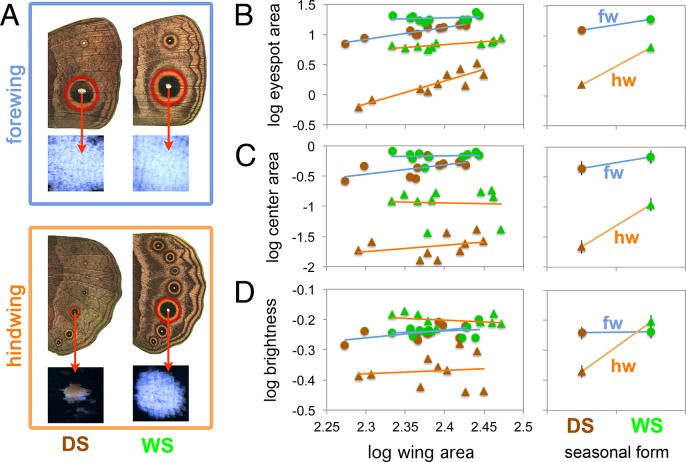
Forewing eyespots display lower levels of plasticity relative to hindwing eyespots. A) Traits measured in this study were eyespot size (mm^2^), center size (mm^2^), and center brightness (reflectance) for Cu1 homologous eyespots on fore and hindwings of DS and WS forms. B-D) Graphs on the left depict the allometric relationship between log trait size (or log brightness) and log wing size. Green and brown symbols represent WS and DS forms respectively. Lines of best fit are blue for forewings, fws, and orange for hindwings, hws. Right-hand graphs depict the estimated marginal means from each eyespot type, evaluated at a wing size of 245 mm^2^. Notice how hindwing eyespots display steeper slopes, e.g., stronger plasticity, relative to forewing eyespots. A significant wing by seasonal form interaction (i.e., significantly different slopes for forewings and hindwings) is present in each case (see Table 1 for test statistics). No plasticity is present in the brightness of forewing eyespot centers (D). Error bars represent 95% CI for the means.

In order to examine whether eyespots on the permanently exposed hindwings and the conditionally exposed forewings exhibit differences in plasticity in response to rearing temperature, we measured the overall size of the large Cu1 eyespot (Fig 1) on the forewing and its serial homologue on the hindwing in *B*. *anynana* female butterflies from cohorts reared at low temperature (producing the DS form) and high temperature (producing the WS form). Because eyespot visibility and conspicuousness may not only depend on overall size, but also on the brightness and size of the UV-reflective white centers [10], which are important deflective targets of attacks by vertebrate predators [9], we also measured the size and brightness of these centers in the two *B*. *anynana* seasonal forms. Having found differences in plasticity across forewing and hindwing serial homologous eyespots, we then investigated the molecular and physiological mechanisms of these differences. We performed temperature-shift experiments to discover the developmental stage that is most sensitive to rearing temperature; measured hormone titer levels and hormone receptor expression at that stage in development to test whether the plasticity was being mediated by variation in either of these factors; and conducted hormone and hormone receptor manipulation experiments to confirm the physiological mechanism controlling differential plasticity across serial homologs. We used a candidate gene approach for the hormone study focusing mostly on ecdysteroids and juvenile hormones as these have often been implicated in the regulation of phenotypic plasticity in insects [18, 19]. We describe the complex physiological mechanism controlling ventral eyespot size and brightness plasticity in *B*. *anynana* and detail the first example of a molecular mechanism that allows serial homologs to exhibit different levels of plasticity along a body axis.

## Results

### Hindwing eyespots display higher levels of plasticity in overall size, center size and center brightness than forewing eyespots

WS ventral eyespots were overall larger than DS ventral eyespots for both forewing and hindwings (Fig 1B and Table 1). However eyespot size plasticity was more extreme for hindwing eyespots relative to forewing eyespots: hindwing eyespots had a steeper slope of area increase with temperature relative to forewing eyespots (Fig 1B, right-hand graph and Table 1). Eyespot center size was also plastic across both wings, with WS eyespot centers being larger than DS centers (Fig 1 and Table 1). However, center size plasticity was again more extreme in hindwing eyespots (Fig 1C, right-hand graph, and Table 1). Thus, both eyespot size and center size are seasonally plastic traits as previously reported [15, 20] but hindwing eyespots show a larger degree of plasticity relative to forewing eyespots, a finding not previously reported.

**Table 1:** F statistics and p-values from analysis of covariance probing for differences in Cu1 eyespot size, eyespot center size, and center brightness between season form (DS and WS forms) and wing (FW and HW). Wing size was used as a covariate. All data were log10 transformed. Brightness of eyespot centers was calculated as the integral of reflectances from 300-750nm, whereas visible brightness was integrated from 400–750 nm. Notice the significant interaction between wing and seasonal form in all analyses, indicating that forewings and hindwings do not respond to rearing temperature in similar ways.

Trait	Factors used in Ancova	F values	p-values	DF (Factor, Error)
Eyespot size	Wing	548.99	<0.001	1, 35
	Form	182.80	<0.001	1, 35
	Wing x Form	60.02	<0.001	1, 35
Eyespot center size	Wing	355.61	<0.001	1, 35
	Form	62.77	<0.001	1, 35
	Wing x Form	22.53	<0.001	1, 35
Center brightness (300–750 nm)	Wing	19.01	<0.001	1, 35
	Form	61.50	<0.001	1, 35
	Wing x Form	59.96	<0.001	1, 35
Center visible brightness (400–750 nm)	Wing	7.20	0.011	1, 35
	Form	48.75	<0.001	1, 35
	Wing x Form	47.58	<0.001	1, 35

Ventral eyespot center reflectance, integrated across 300-750nm of the light spectrum, was not plastic for forewing eyespots, but was significantly plastic for hindwing eyespots (Fig 1D and Table 1). Forewing eyespot centers were bright irrespective of rearing temperature (reflectances ~ 0.6), whereas hindwing eyespots were bright in WS forms but dull in DS forms. Similar results were obtained across the narrower visible light spectra (from 400 to 750 nm; Table 1). Since results for the two spectral ranges were comparable, we subsequently used a grey value measurement (K value, “blackness”) obtained from B&W photos of eyespot centers as a proxy for eyespot center reflectance (see M&M). These K values had a highly significant negative correlation (Pearson’s coefficient = -0.67; p < 0.001) with the whole reflectance values (integrated from 300–750 nm). In summary, eyespot center reflectance exhibits different levels of phenotypic plasticity across forewing and hindwing serial homologs, just as eyespots size does.

**Fig 2:**
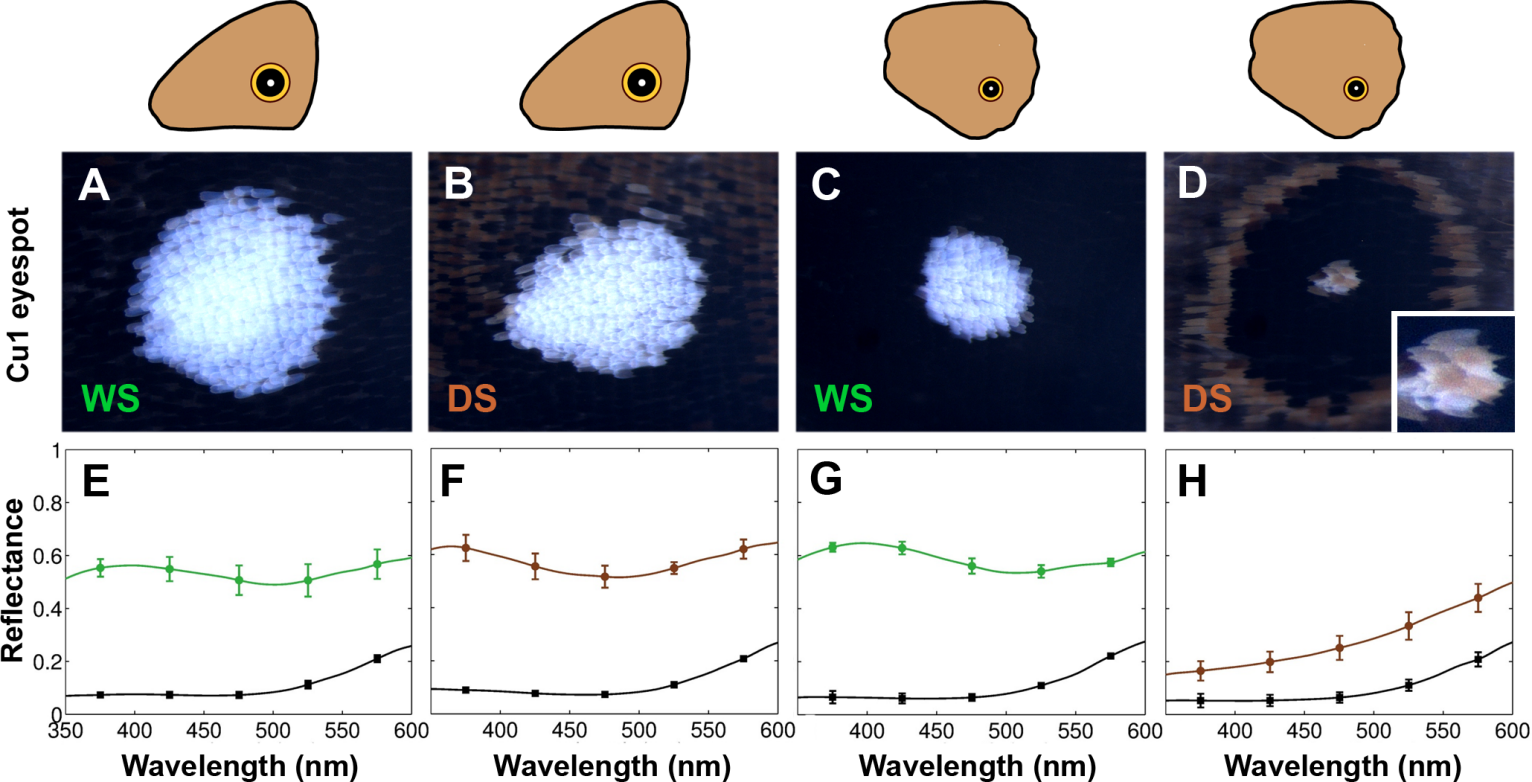
Plasticity in eyespot center brightness is due to variation in pigment deposition in the white central scales. Epi-illumination microscopy and reflection measurements for Cu1 eyespot centers in forewings (FW) and hindwings (HW) of both WS and DS forms. A) WS FW; B) DS FW; C) WS HW; D) DS HW. E-H) Reflection measurements of corresponding eyespot centers before (colored lines) and after (black lines) application of silicone oil.

To examine the cause of the differences in reflectance between DS and WS hindwing eyespot centers, we examined those centers closely. WS centers were bright white, as were DS forewing centers, whereas DS hindwing scales were yellowish in appearance (Fig 2A–2D). To determine whether the white and yellow colors were structurally generated or due to pigments, we used refractive index matching analyses. Structural colors in butterfly scales are generated through light scattering from wing scale nanostructures made of chitin that have a refractive index difference from air [21]. Therefore, by soaking the wing in silicone oil with a refractive index (n = 1.4) similar to that of chitin (n = 1.56) [22], we can reduce the refractive index contrast, and suppress light scattering from chitin nanostructures. As a result, structural colors should disappear or diminish, allowing us to visualize pigmentary colors alone. For the bright white scales we observed significant changes in the magnitude of the reflectance spectra before and after soaking the wing scales in silicone oil (Fig 2E, 2F and 2G). This indicates that the white color is primarily a structural color. For the yellowish scales, however, we observed a smaller change in the magnitude of the reflection spectrum, suggesting that this yellow color results from light absorption by pigments (Fig 2H). In addition, the yellow centers coated in oil had lower values of light transmission (i.e., the scales were less transparent) than the bright white scales, due to more pigment deposition (S2 Fig). This pigment was preferentially deposited in the scale ridges (see S1 File). So, the color difference between DS and WS scales on the hindwing is primarily due to additional pigments being deposited in the DS scales in response to low rearing temperatures.

### Plasticity of (hindwing) wing patterns is determined by temperature during the wandering stage of larval development

**Fig 3:**
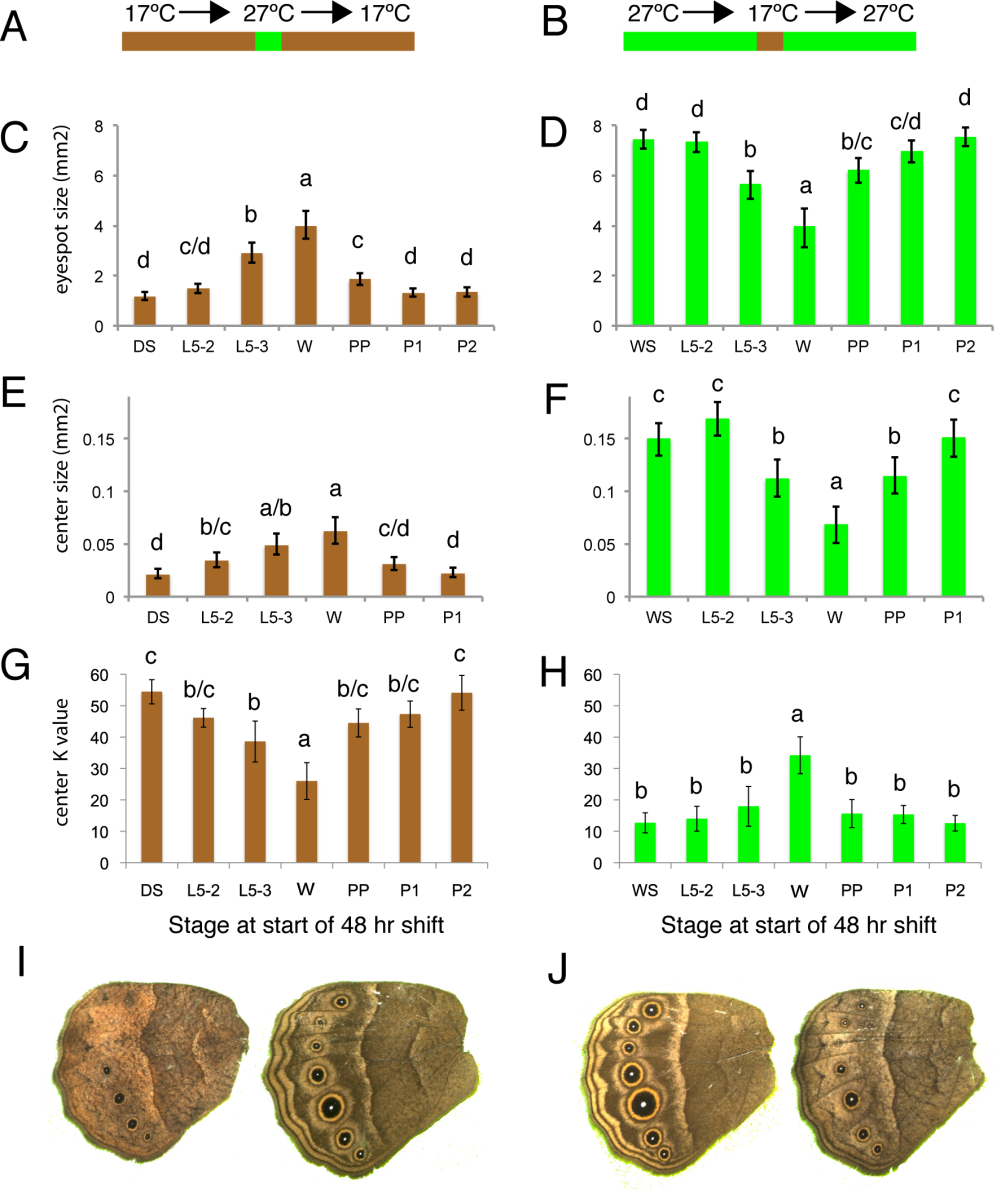
Temperature shift experiments point to the wanderer stage of development as the most sensitive stage regulating ventral HW eyespot size and brightness. A, B) Animals were reared at either 17°C (brown) or 27°C (green) for most of their development with the exception of a single 48hr window where they were reared at the other temperature. C, D) Estimated eyespot size or eyespot center size (E, F) for wings with constant area of 250 mm^2^. G, H) Eyespot center K value (measure of darkness) of animals shifted at different stages during their development. DS: non-shifted Dry Season animals; WS: non-shifted Wet Season animals; L5-2: animals shifted at start of stage 2 during the 5^th^ instar (see Table 2 for stage description); L5-3: at start of stage 3 during the 5^th^ instar; W: at start of wanderer stage; PP: at start of pre-pupal stage; P1: at start of pupal stage 1; P2: at start of pupal stage 2. C, E, G) Most of development happened at 17°C. D, F, H) Most of development happened at 27°C. I) Representative DS control individual (left) versus DS shifted to higher temperatures at wanderer stage. J) Representative WS control individual (left) versus WS shifted to lower temperatures at wanderer stage. Error bars = 95% CI of the means. Experimental groups labeled with the same letter: “a”, “b”, “c” or “d” are not significantly different from each other, whereas groups labeled with a different letter are.

In order to explore when during development eyespot size, center size, and center brightness were responding to rearing temperature we performed a series of temperature shift experiments. Phenotypes of individuals reared constantly at 17°C and 27°C were compared with those of individuals reared primarily at one temperature, but shifted for a narrow window of 48hrs to the other rearing temperature (Fig 3A; See Table 2 for description of developmental stages used for the switch). Here we concentrated on measuring hindwing eyespots only, as these were the most plastic.

**Table 2:** Developmental staging used in current study. Total larval development from 5^th^ instar ecdysis to pupation for female *B*. *anynana* takes approximately 8 days at 27°C (WS) and 20 days at 17°C (DS). Total pupal development until adult emergence takes an average of 6.4 days for WS and 16 days for DS females. The bright green wanderer stage occurs, on average, on day 6 for WS forms, and on day 17 for DS forms. Temperature shifts, hemolymph collection, and injections were all performed at 2pm of noted day. Note the difference in stage nomenclature for temperature shifts during the pupal stage and hormone titer quantification (equivalent % development time).

Developmental stage abbreviation	Description	Staging: days from 5^th^ instar ecdysis or from pupation.	% larval or pupal development
L5-2	5^th^ Larval stage 2	2 (WS)	25% (WS)
		5 (DS)	25% (DS)
L5-3	5^th^ Larval stage 3	4 (WS)	52% (WS)
		10 (DS)	50% (DS)
W	Wanderer stage (bright green)	6 (WS)	77% (WS)
		17 (DS)	84% (DS)
PP1	Pre-pupal stage 1	7 (WS)	90% (WS)
		18 (DS)	89% (DS)
PP2	Pre-pupal stage 2		
		19 (DS)	94% (DS)
P1 (for temp shifts)	Pupal stage 1	1 (WS)	13% (WS)
		2 (DS)	10% (DS)
P1 (for hormone quantification)	Pupal stage 1	1 (WS)	13% (WS)
		3 (DS)	16% (DS)
P2 (for temp shifts)	Pupal stage 2	2 (WS)	31% (WS)
		3 (DS)	16% (DS)
P2 (for hormone quantification)	Pupal stage 2	2 (WS)	31% (WS)
		5 (DS)	29% (DS)

All three traits became larger and brighter (i.e., lower K values) when animals were reared for 48 hrs at high temperatures starting at the wandering stage of development (Fig 3C, 3E and 3G). The opposite effect (i.e., eyespots becoming smaller/duller) was observed when animals were reared at low temperatures starting at this same stage (Fig 3D, 3F and 3H). Significant, but smaller effects on eyespot size and eyespot center size and brightness were also seen at the two flanking developmental time points to the wanderer stage, late larvae (L5-3) and pre-pupal (PP) stages, but not at the earlier larval stages (L5-2, with one exception) nor later pupal stages (P1 or P2) of development. We conclude, therefore, that ventral eyespot size and brightness plasticity is primarily being determined during a relatively narrow window of development centered on the wandering stage.

### 20-hydroxyecdysone titers are different across seasonal forms at the wandering stage of development

We then explored whether insect hormones could be mediating eyespot size and brightness plasticity. We measured the titers of three common insect hormones, 20-hydroxyecdysone (20E), ecdysone, and juvenile hormone, at the wandering stage (in bright green larvae), as well as at two time points before and after this stage, in females from the two seasonal forms. The wanderer stage in the DS forms lasts on average 43.5 hours (~ two days), with the first day wanderers having a darker green appearance relative to the bright green appearance of 2^nd^ day wanderers (the stage measured in both forms). The wanderer stage in the WS form lasts on average 25.65 hours (~1 day). The onset of the wandering stage is around 11.30 am for DS animals and 11 pm for DS animals. By sampling wanderers at 2pm in their bright green stage, we sampled animals at comparable 60.9% (DS) and 59.8% (WS) of the wanderer stage. The pre-pupal stage in DS forms lasts on average 41.83 hrs (~2 days), whereas it lasts 20.83 hrs (~1 day) in WS forms. The titer measurements taken at 2pm from day 1 pre-pupae (PP1) correspond to 17% (DS) and 62.5% (WS) of pre-pupal development, whereas the measurements taken from day 2 DS pre-pupae (PP2) corresponds to 74% of pre-pupal development. We therefore compared the WS PP1 titers with DS PP2 titers.

**Fig 4:**
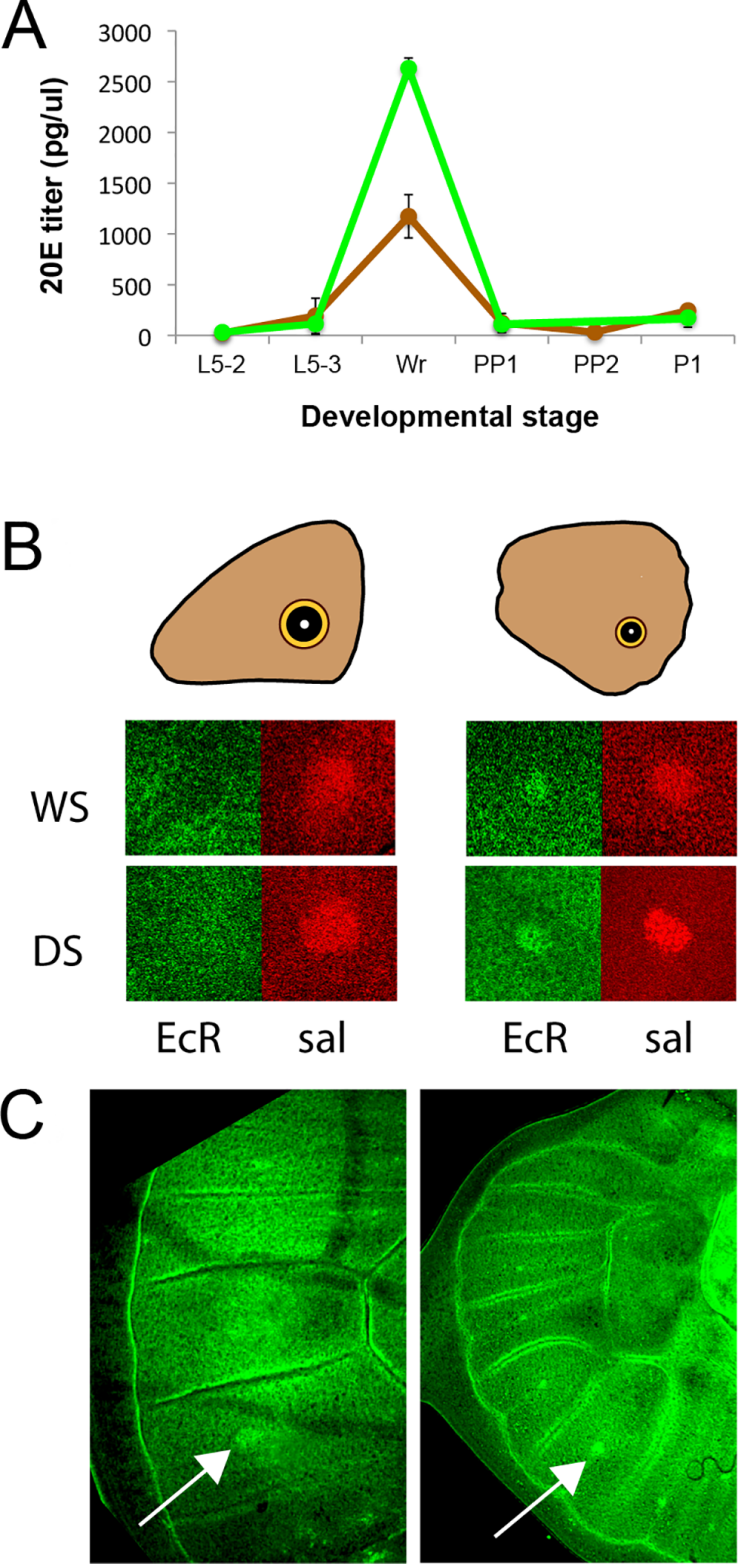
20E hormone titers are higher in WS forms at the wanderer stage of development and the 20E receptor (EcR) is present in hindwing but absent from forewing eyespot centers at that stage. A) Titers of 20-hydroecdysone (20E) at several stages of larval, pre-pupal and pupal development. DS forms = brown line; WS forms = green line; Stages described in Table 2. Error bars correspond to 95% CI for means. B) During the wanderer (W) stage of development EcR (green) is expressed in hindwing eyespot centers of both seasonal forms (WS, DS) but not in forewing eyespots, whereas a second eyespot-associated protein, spalt (sal; red), is expressed in all eyespot centers. C) Earlier during the 5^th^ instar, however, EcR is expressed in both forewing (left) and hindwing (right) eyespot centers on the ventral surface. Arrows point to the Cu1 eyespot.

We focused our attention on 20E because we observed that titers of this hormone were significantly more elevated in the WS forms relative to DS forms at the wandering (W) stage of development (F = 60.035, p<0.001) (Fig 4A). There were no differences in 20E hormone titers at the other stages examined (L5-2: F = 0.511, p = 0.501; L5-3: F = 0.584, p = 0.474; PP1/PP2: F = 0.049, p = 0.832; P1: F = 1.503, p = 0.266) (Fig 4A). In summary, rearing temperature alters the titers of 20E during one of the three previously identified temperature-sensitive points in development for the regulation of eyespot size and brightness plasticity. This indicates that temperature could be regulating plasticity in these traits via regulation of 20E titers.

### The different levels of plasticity in forewing and hindwing eyespots are associated with the differential expression of the ecdysone receptor (EcR) in these eyespot centers

Next we examined whether cells in the eyespot field are potentially sensitive to titers of 20E circulating in the hemolymph during the bright green wandering stage of development. Cell sensitivity to 20E signaling requires the expression of the 20E receptor, the Ecdysone Receptor (EcR). We used an antibody developed against the common isoform of EcR of *Manduca sexta* [23], to localize EcR in *B*. *anynana* wing discs as had been previously done for *Junonia coenia* [24]. EcR was expressed at low levels throughout the ventral wing epidermis, but it was expressed at higher levels in the central cells of hindwing eyespots of both seasonal forms (Fig 4B). EcR expression, however, was absent from the center of forewing eyespots during the bright green wandering stage (Fig 4B). To investigate whether EcR expression was temporally being modulated in forewing eyespots we examined earlier 5^th^ larval instars and observed the presence of EcR across all eyespot centers, including forewing eyespots (Fig 4C). These data suggest that forewing and hindwing eyespots may exhibit different sensitivities to 20E signaling because their central cells differentially express EcR during the critical temperature-sensitive stage of development.

### Disruption of EcR signaling during the wandering stage of development alters eyespot size, center size, and center brightness predominantly in hindwing eyespots

**Fig 5:**
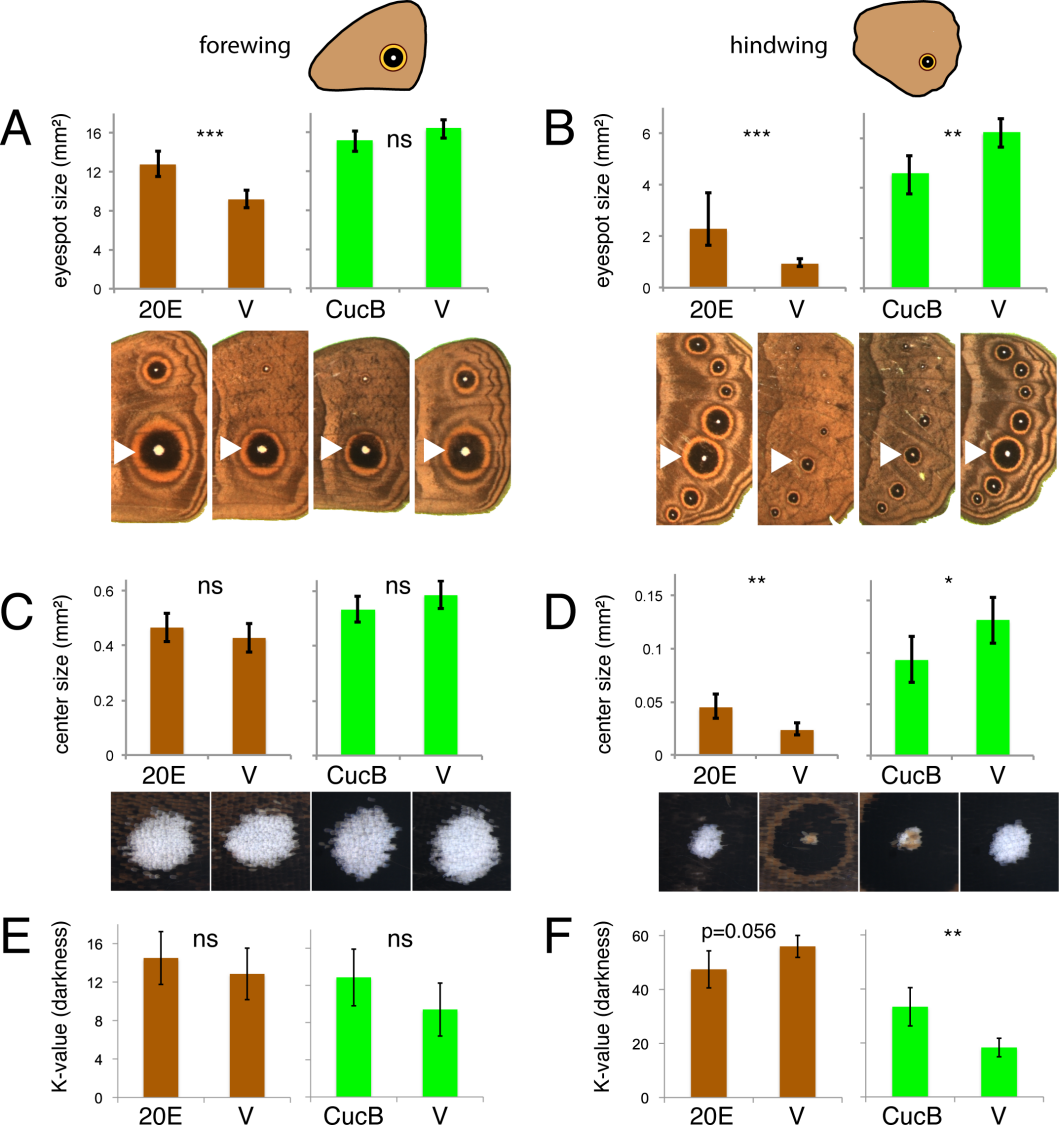
Manipulations of ecdysone signaling during the wanderer stages of development alters hindwing eyespots more extensively than forewing eyespots. Injections of 20-hydroxyecdysone (20E) and vehicle (V) were performed in DS forms (brown). Injections of Cucurbitacin B (CucB) and vehicle were performed in WS forms (green). Measurements of eyespot size (A, B), eyespot center size (C, D) and eyespot center darkness (K value) were performed in forewing eyespots (A, C, E; left graphs) and hindwing eyespots (B, D, F; right graphs). Error bars = 95% CI of means. Significant differences between treatments are represented by asterisks: *, p < 0.05; **, p < 0.01, ***, p < 0.001, ns = non significant. Values for trait size in A-D are evaluated at a wing area of 227 mm^2^.

To test whether 20E signaling during the wandering stage of development contributes (differentially) to eyespot size, center size, and center brightness plasticity between forewing and hindwing eyespots, we manipulated 20E signaling using 20E injections into bright green wanderers and measured the resulting phenotypes. Because 20E may have direct effects on eyespots (via the EcR receptor expression in eyespots) and indirect effects on eyespots (via potential stimulation of other hormonal signaling systems) [25], we also tested whether EcR-mediated signaling was directly contributing to trait variation using injections of an EcR signaling antagonist, cucurbitacin B. This molecule binds the EcR receptor and prevents the 20E ligand from binding it [26]. Injections of 20E into DS bright green wanderers, who normally display lower titers of this hormone, led to significant increases in eyespot size relative to vehicle-injected individuals in both forewings and hindwings (Fig 5A and 5B; left-hand graphs; forewing: F_1,37_ = 21.916, p<0.001; hindwing: F_1,37_ = 30.881, p<0.001). However, increases were more pronounced (140% versus 39% of the eyespot size of vehicle-injected animals for animals with the same wing size) in hindwings relative to forewings. Conversely, injections of cucurbitacin B into WS wanderers, who normally display higher titers of 20E, led to significant decreases in eyespot size relative to vehicle-injected individuals but only in hindwings (Fig 5A and 5B; right-hand graphs; forewing: F_1,37_ = 3.363, p = 0.075; hindwing: F_1,37_ = 12.636, p = 0.001). Injections of 20E also led to a significant increase in eyespot center size and a nearly significant increase in eyespot center brightness (decrease in K values), but only in hindwings (Fig 5C–5F; left-hand graphs; forewing center size: F_1,37_ = 1.065, p = 0.309; hindwing center size: F_1,37_ = 13.971, p = 0.001; forewing center brightness: F_1,38_ = 0.712, p = 0.404; hindwing center brightness: F_1,38_ = 3.879, p = 0.056). Conversely, injections of cucurbitacin B into WS wanderers led to significant decreases in eyespot center size and center brightness (increase in K value) relative to vehicle-injected individuals but, again, only in hindwings (Fig 5C–5F; right-hand graphs; forewing center size: F_1,37_ = 2.595, p = 0.116; hindwing center size: F_1,37_ = 5.539, p = 0.024; forewing center brightness: F_1,38_ = 2.625, p = 0.113; hindwing center brightness: F_1,38_ = 14.066, p = 0.001). These results indicate that eyespot size, eyespot center size, and center brightness are differentially regulated in forewings and hindwings by 20E signaling during the wandering stages of development: forewing eyespots responding less strongly to 20E signaling manipulations relative to hindwing eyespots.

## Discussion

All eyespots appear to share a core gene regulatory network that evolved once, roughly 90 million years ago, near the base of the nymphalid clade of butterflies [27]. This network, originally deployed on the hindwing later moved to homologous wing positions on the forewing creating serial homologous forewing and hindwing eyespots [7, 8]. In *B*. *anynana*, who lives in seasonal environments, visible hindwing eyespots came under alternating types of selection to serve a predator-deflective function in the WS cohort and a cryptic function in the DS cohort, and evolved size and brightness plasticity in response to temperature cues. Hidden (but conditionally displayed) forewing eyespots, however, likely maintained the same predator-deflection or sexual signaling function in both seasons and evolved a fairly fixed and conspicuous phenotype, irrespective of temperature cues. The homologous eyespot gene regulatory network evolved, thus, different levels of plasticity along the body axis.

Our work proposes that differential expression of EcR across forewing and hindwing serial homologs coupled with temperature-induced changes in 20E titers at a critical stage during development is causing the different levels of phenotypic plasticity across these homologs. We showed that rearing temperature induces changes in eyespot characteristics (size and scale brightness) in *B*. *anynana* primarily during the wandering stages of development. Temperature affects eyespot phenotypes by altering the titers of 20E hormones at a critical stage during development, where higher temperatures lead to higher hormone titers. These hormones circulate within the body, but only those cells that express the hormone receptor, EcR, are able to use hormone titers as a cue to alter their developmental fate. Both WS and DS forms express EcR in cells at the center of future hindwing eyespot patterns at the bright green wandering stage of development. However, EcR is not expressed in forewing eyespots at this stage. This allows forewing serial homologs to be insensitive to fluctuating 20E hormones, while allowing hindwing eyespots to be sensitive to these hormones. In support of this mechanism, injections of 20E and of a EcR receptor antagonist (CucB) at the wandering stage affected the size and brightness of hindwing eyespot centers to a greater extent to that of forewing eyespots. We propose, thus, a novel physiological and molecular mechanism underlying variation in phenotypic plasticity across these serial homologs: variation in 20E signaling caused by variation in the expression of EcR across serial homologs at a critical stage of development. The differential expression of EcR between forewings and hindwings may be related to differences in developmental rate between these two wings, with hindwings often lagging behind forewings, but this will need to be tested with a finer sampling of EcR expression around the developmental stage examined here.

Molecular mechanisms underlying differences in plasticity across different body parts were previously examined in *Drosophila*, *Ontophagus*, *and Trypoxylus* beetles regarding nutritional plasticity, but appear to be different from the mechanism proposed here [28–30]. Nutritional plasticity refers to changes in adult body size in response nutrition levels experienced during development. Changes in levels of plasticity across body parts are mediated by the Insulin/IGF-signaling (IIS) pathway [31]. In flies, for instance, where the molecular mechanisms have been probed in greater detail, wings and maxillary palps are more sensitive to insulin signaling (are more nutritionally plastic) than genitalia [29]. This variation in plasticity levels is due to variation in expression levels of the transcription factor Forkhead Box O (FOXO), acting downstream of the insulin receptor, in the different body parts [29]. Our work identifies a different signaling pathway, 20E signaling, and a different molecular candidate, EcR, for the differential regulation of (seasonal) plasticity across body parts or, in this case, serial homologs.

Given our proposed model for the involvement of 20E signaling in the regulation of eyespot size and brightness plasticity, and given the absence of EcR from the center of forewing eyespots during the wandering stage, it is unclear why there was a significant change in forewing eyespot size in response to 20E injections during this stage. The most likely possibility is that 20E injections are affecting hindwing eyespots via a direct mechanism, and both forewing and hindwing eyespots via an indirect mechanism. The indirect mechanism could involve the 20E injections regulating other hormonal systems that subsequently regulate eyespot genes independently of EcR signaling [25]. This would explain why directly interfering with EcR-mediated signaling using CucB, which directly binds the receptor and prevents it from binding the 20E ligand [26], showed only hindwing-specific effects in eyespot size, whereas injections of 20E, showed effects on both forewing and hindwing eyespots (Fig 5A). The difference in the eyespot response to these two types of signal modulation indicates that injection of hormones, as often done in the field of insect endocrinology, should not be used alone to infer that a particular hormonal signaling pathway is affecting the normal development of a trait [25]. Extirpation of hormone production organs [32, 33], or down-regulation of the specific hormone receptor [30] are also necessary.

Overall, our results indicate that the regulation of ventral eyespot size, center size, and brightness plasticity is occurring during a short window of development centered around the bright green wandering stage when temperature sensitivity, hormone titer differences, hormone receptor expression in critical eyespot signaling cells, and sensitivity to EcR signaling, all co-occur at the same time. These results challenge previous conclusions on the regulation of ventral eyespot size plasticity in *B*. *anynana*. In particular, earlier work proposed that 20E hormone titer differences during the pupal period were responsible for ventral eyespot size plasticity [34, 35], whereas we now show that titer differences during the wandering stage of larval development appear to be the primary regulators of this plasticity. An original set of temperature shift experiments (where animals were only shifted once and did not return to the original temperature) identified, as we did, the late larval stages of development as the critical sensitive period for the regulation of ventral eyespot size plasticity [36]. However, these experiments were then followed by the measurement and manipulation of hormone titers (of genetic mimics, not the actual seasonal forms), in the pupal stage not the late larval stage [34, 35]. Titer differences observed around 30–50% of the pupal stage between the actual DS and WS forms [34, 37] were later shown to primarily control development rate, not eyespot size [38].

More recent work also attempted to investigate the molecular mechanisms explaining differences in levels of eyespot plasticity between eyespots on different wing surfaces by measuring responses to 20E injections in *B*. *anynana*, but was inconclusive [39]. This work focused on earlier pupal stages (3–16% of development), which show no significant differences in 20E titers between DS and WS forms [37]. The authors concluded that despite injections of 20E producing more variation in size of ventral eyespots relative to dorsal eyespots, the different levels of response observed could not be explained by eyespot-specific variation in EcR expression, which was observed across both ventral and dorsal eyespots at that time in development [39].

Our work builds on all this previous work by focusing on the same candidate hormonal signaling system, 20E, on the same previously identified temperature sensitive period [36], on the same identified hormone receptor [24, 39, 40], but expands it by identifying previously undetected 20E hormone titer differences between the seasonal forms during the late larval stage (the wandering stage), and identifying the mechanism that is likely driving ventral eyespots size (and eyespot brightness) plasticity of WS and DS seasonal forms, as well as the mechanism responsible for differences in plasticity across forewing and hindwing eyespots.

It is important to note that while eyespot size regulation in *B*. *anynana* is taking place during the wandering stages of development, previous work in *Junonia coenia* and *Araschnia levana* butterflies showed that differences in 20E sensitivity during the pupal stage was controlling color polyphenism in these species [32, 33]. It is possible that the different developmental times used by these species to regulate pattern element size and color plasticity are constrained around the period in development when these two types of trait are being differentiated. In *B*. *anynana*, the over-expression and down-regulation, during larval development, of another developmental gene found in eyespot centers, *Distal-less*, affects eyespot size, whereas the ectopic expression of this same gene during pupal development, when the gene is associated with black pigmentation, alters pigment development [41]. If the developmental control of size and color is happening at two distinct developmental stages in *B*. *anynana*, the environmental regulation of these traits may need to coincide with those same periods in development.

### EcR as a key locus in the regulation of plasticity

Our data suggests that the transcription factor EcR plays a key role in differentiating serial homologous eyespots in regards to their level of plasticity. This role is both similar and different to the role of other transcription factors, such as Ultrabithorax (Ubx), in differentiating serial homologs, such as insect wings, across thoracic segments [42, 43]. The similarities involve the differential expression of these genes in the serial homologs that will become individuated from the others, e.g., the expression of Ubx and EcR in hindwings or hindwing eyespots, but not in forewings or forewing eyespots, respectively. The differences involve Ubx leading to the permanent (fixed) modification of the wing network where it is expressed, whereas EcR leading to plastic modifications across individuals of the hindwing eyespot network, where it is expressed. The key property that distinguishes EcR from Ubx is that EcR needs to bind the ligand 20E to become transcriptionally active, and 20E titers are varying with environmental cues. So, by having recruited an environmentally-induced asymmetric cue to modify its output, the eyespot gene network can produce two alternative phenotypes at the same position in the body (in alternative generations) whereas the wing network can’t. Once genes from the eyespot network have evolved sensitivity to the EcR transcription factor, e.g., once they have evolved ecdysone response elements (EcREs) in their cis-regulatory elements [44], the easiest way for subsets of serial homologs to modify their level of plasticity is for mutations to either disrupt or co-opt the expression of EcR to these serial homologs. This leads to the evolution of EcR as a key locus in the regulation of plasticity across serial homologs.

### Implications of this work for the field of evolution of plasticity

The results described above present a static view of the regulation of a complex phenotype, phenotypic plasticity, in serial homologous traits of a single species, *Bicyclus anynana*. However, these results don’t address how such a complex system actually evolved. To progress in this area, future comparative work should map origins of the plasticity-enabling molecular/physiological components on a phylogenetic tree of nymphalid butterflies where we previously mapped the origin of eyespots [6]. This includes mapping: 1) The origin of the fluctuating 20E titers during the wanderer stages of development with different rearing temperatures; 2) The origin of expression of EcR in each of the eyespot serial homologs; 3) The origin of sensitivity by eyespot gene network members to 20E signaling, and finally 4) The origin of eyespot plasticity in response to environmental temperature (the complete complex trait). By documenting above how ventral eyespot size/brightness plasticity is mechanistically controlled in one species, we can now begin to explore how this complex physiological/molecular system originated and evolved.

## Materials and Methods

### Butterfly husbandry

*B*. *anynana* butterflies were reared in two walk-in climate rooms at 17°C and 27°C, leading to the development of the dry and wet season forms, respectively. They were also reared at 80% relative humidity, and 12:12 hours light:dark cycle. Adults were fed banana while larvae were fed young corn plants.

### Eyespot and eyespot center size measurements

*B*. *anynana* female adults from each season (previously stored at -20C) were dissected and imaged using equipment described in Tong *et al*. (2012). Images were acquired at 3X magnification for entire wings and 75X for eyespot centers. Area measurements for forewings (right only), hindwings (right only), individual eyespots (including outer gold ring), and pupils (white center) were calculated using ImageJ (NIH, v1.45s). We used a freehand tool to outline wing area and eyespot centers for measurements in Fig 2, and in subsequent analyses used a threshold tool in Image J and the magic wand tool in Photoshop CS3 to outline wing area and eyespot centers, respectively. Eyespot area was measured by fitting an ellipse to the outer gold ring of each eyespot in Image J.

### Reflectance measurements

Wings (right only) were attached to a glass slide. A broadband white light source (Xenon arc lamp) was used to illuminate the pupils (white scales at focus). A UV objective lens (Newport U-27X) focused light into a 70um diameter circular spot on the sample. Reflected light was collected by the same objective lens and then coupled by an additional UV thin lens into a multimode fiber. The other end of the fiber was connected to a spectrometer (Ocean Optics HR-2000+). The spectrum of the reflected light was recorded, and normalized to the reflection spectrum from a white diffuse reflectance standard (WS-1, Ocean Optics). Measurements were taken from three different areas of each eyespot center, and these measurements were repeated for ten different wings of each sex and seasonal form. Eyespot center darkness was also quantified by K scores in Photoshop CS3: 1) We used the magic wand tool to select the area containing the eyespot center in digital images taken under the same lighting conditions; 2) averaged the color in this area using the Filter/Blur/Average series of menu commands; 3) placed thumbs of all images in the same plate; 4) converted the composite image to grayscale; 5) used the levels tools to adjust image brightness and contrast; and 6) used the color picker to measure the K value of the pupil area. These K values were highly correlated to the spectrometer measurements taken from the same specimens (see text).

### Refractive index matching experiments

To investigate whether color differences observed in white scales were due to structural changes, we used silicone oil (Fisher Chemicals) with refractive index of 1.402 to suppress light scattering from the scales with a refractive index of 1.56±0.01 [22]. We placed a droplet of silicone oil on the white scales, let the oil penetrate into the samples and fill the air voids between the cuticle’s materials during 2–3 hours, and measured the reflectance spectra before and after applying oil. Each reflectance spectrum was averaged over 2 samples.

To identify the location of the light scattering nanostructures in the white scales, we extracted and observed single wing scales at high magnification (100x). Scales were extracted by pressing the white pupil softly against a glass slide (using a Texwipe polyester swab) until the wing scales detached. Double-sided adhesive tape was used to collect and affix the wing scales onto another glass slide. Images for single scales were taken both in reflection mode and in transmission mode before and after the application of silicone oil.

### Hormone titer measurements using ultra pure liquid chromatography and mass spectrometry (UPLS/MS)

#### Hemolymph collection

A small puncture was made to the first abdominal proleg of individual larvae, wanderers, and pre-pupae, or lateral posterior region of the fifth abdominal segment of individual pupae, at 2 pm, and 10 μl of hemolymph were collected using a pipet. Hemolymph collections were taken from WS and DS female larvae/pupae at five developmental time points: L5-2, L5-3, wanderer (bright green), pre-pupae (day 1) for DS and WS individuals, and pre-pupae (day 2) for DS individuals, and at the P1 pupal period (N = 4 per time point per seasonal form; see Table 2 for details). The time of day of hemolymph collection was held constant (2pm) in order to avoid the confounding effects of daily fluctuations in hormone titers [45], and because development rate is known to be adjusted to light cycle during the wandering stage in other lepidopterans [46]. To calculate the stage of development (in %) correspondent to the 2pm sampling of WS and DS larvae during their wandering and pre-pupal stages, multiple fifth instar larvae (N > 20) were placed in individual containers in front of a camera and were photographed every 5 minutes using time-lapse photography. We estimated total developmental time for each of these stage by capturing the moment when larvae stopped feeding on food and became wanderers, the moment these wanderers hung themselves via a silk pad to become pre-pupae, and the moment these animals became pupae, for each of the seasonal forms. The hemolymph from each individual was then placed in a solvent solution of methanol/iso-octane (1:1, v/v), with a hemolymph–solvent ratio of 1:10 (v/v). The mixture was vortexed for 20 seconds and then stored at -80°C until sample extraction. This sample preparation followed an established protocol [47].

#### Hormone extraction

We added 900 μl of HPLC grade water to the 100 μl sample of 10 μl of hemolymph + 45 μl methanol + 45 μl iso-octane and then vortexed the solution. For the 20ul hemolymph samples we used 90 μl methanol, 90 μl iso-octane, and 800μl of water. We then used Waters Oasis HLB SPE cartridges (1cc cartridge Part # 186000383) in an extraction manifold (Waters Part# WAT200677) to separate the hormone-containing methanol layer from the iso-octane layer. Before adding our hormone samples, we primed the HLB cartridges with 1000 μl methanol and then 1000 μl HPLC grade water. We then added the 1000 μl hormone-containing sample to the HLB cartridge, but did not collect the elution. Then, we added 1000 μl of HPLC grade water to the HLB cartridges and also did not collect the elution. We repeated this step three times to wash the cartridge. We then added 50 μl of methanol to the HLB cartridge to elute the hormone from the cartridge. The elution was placed at -20°C until hormone measurement.

#### Hormone titer measurements using UPLC/MS

Hormone titers of samples displayed in Fig 4A were measured by the W.M. Keck Foundation Biotechnology Laboratory at Yale University using a Perkin Elmer Flexar Ultra High Pressure Liquid Chromatography System coupled in-line to a 4000 Q-TrapLCMS/MS system. The hormones were separated utilizing an Agilent Technologies ZORBAX Eclipse XDB-C18 (3.0 x 100mm, 3.5 micron pore size) column (p.n. 961967–302), coupled to an analytical Phenomenex SecurityGuard trap (C18, 4 x 3.0mm). The column and trap were kept at 40°C. Briefly, the samples were eluted at a flow rate of 500 μL/min using a methanol:water-based mobile phase which contained 0.1% formic acid. A blank injection of 100% methanol was run after each sample injection to ensure no carry over. Optimization of the differential potential (DP) and collision potential (CE) were carried out utilizing direct injection of standard insect hormones purchased from Sigma-Aldrich (CAS# 0005289747). A mixture of the standard was then carried out to determine the best gradient to use for the sample runs. Standard curves of mixed standards were used to calibrate specific transitions for each of the hormone. We monitored three transitions for 20E, but only one transition (the one which provided the best detection response) was utilized in our quantitation measurements. All transitions eluted between 1.5 to 3.2 min in our 6 min gradient run. Caffeine was used as an external control to ensure instruments data acquisition stability and reproducibility for our specific gradient and instrument parameter settings. Source and gas conditions were optimized based on the standard direct injection runs. Data were acquired on the 4000 QTRAP instrument utilizing Analyst 1.5.2. and the collected raw data were processed utilizing Multiquant software (v. 2.0).

### Ecdysone receptor immunostainings

To determine the location of the ecdysone receptor expression in wings of WS and DS butterflies, immunostainings were performed following the protocol of [48]. The wing discs were dissected from late larvae and wanderers. Monoclonal (mouse) antibodies raised against a *Manduca sexta* EcR peptide shared across all EcR isoforms (Developmental Studies Hybridoma Bank, #10F1;[23]) were used at a concentration of 1:5. Guinea pig polyclonal anti-Spalt antibodies were used as a positive control of eyespot specific expression at a concentration of 1:20,000 [49]. Goat anti-mouse (Molecular Probes, #A-11001), and goat anti-Guinea pig (Molecular Probes, #A11076) were used as secondary antibodies at a concentration of 1:200. The wings were mounted with ProLong Gold (Invitrogen, Carlsbad, CA, USA). Images were captured on a Nikon 90i microscope with NIS-Elements software (Nikon Instruments, Mellville, NY, USA). Serial *z-axis* optical sections were also performed in the eyespot region using both LSM 510 META and LSM 710 confocal microscopes (Carl Zeiss, Jena, Germany) in order to distinguish dorsal from ventral EcR expression. At least three biological replicates were obtained for each immunostaining.

### Hormone injections

Female bright green wanderers of the two seasonal forms were injected with 3 μl of 2000 pg/μl of 20E (6000 pg total) (Sigma- Aldrich) or 3 μl of vehicle (1 ethanol:9 saline solution), or with 2 μl of 5600 pg/ul of cucurbitacin B (10,200 pg total) (Sigma- Aldrich) or 2 μl of vehicle (1 ethanol:9 saline solution). All solutions were stored at -20°C. The injections were done using a Hamilton syringe (10 μl 700 series hand fitted microliter syringe with a 33 gauge, 0.5 inch needle). The injection site was on the dorsal surface in between the integument of the second and third thoracic leg after the larvae had been chilled for 1 hour on ice.

### Statistical analyses

Eyespot size and eyespot center size were compared across seasonal forms or treatments using analyses of covariance (ANCOVA), where wing area was used as a covariate. Eyespot center brightness and hormone titers were compared across seasonal forms or treatments using analyses of variance (ANOVA). All analyses used the GLM procedure in SPSS Statistics (version 19). Data was power transformed, when necessary, to meet homogeneity of variance criteria (as determined by a Levene’s test). Reflectance spectral curves (measured between 300–750 nm of the light spectrum) were obtained for the Cu1 eyespot centers on each wing. GLM analyses were performed on the integral of the area under those curves, using seasonal form as a fixed factor. Pair-wise comparisons, using a Bonferroni correction for multiple comparisons, were used to detect which developmental time switch points produced significant differences in eyespot traits in the temperature-shift analyses. Graphs were made in Microsoft Excel (version 14.3.7 for the Mac) and Adobe Illustrator CS3 using reverse transformed data (when applicable).

## Supporting Information

S1 Fig An example of conditional display of forewing eyespots in a species of nymphalid, *Hipparchia fidia*, before (left panel) and after disturbance (right panel). This behavior appears to be especially common in DS forms of *B*. *anynana* in the field (M. de Jong, pers. comm.).
(JPG)

S2 Fig Reflection and transmission images and measurements of white scales from HW Cu1 eyespot centers. A) Microscopy images of single white scales under epi-illumination and transmitted illumination before and after silicone oil application. Black arrow across all images denotes a ridge where light is mostly reflected. B) Transmission measurements of WS (green line) and DS (brown line) white scales of HWCu1 eyespot after application of silicone oil. Error bars represent the standard error of the mean.
(JPG)

S1 File Pigmentation is localized in scale ridges. (DOCX)
