# Supplementary figures and images for: Differential Expression of Ecdysone Receptor Leads to Variation in Phenotypic Plasticity across Serial Homologs
Source: PLoS Genet. 2015 Sep 25;11(9):e1005529. doi: 10.1371/journal.pgen.1005529 (PMC4583414; doi:10.1371/journal.pgen.1005529)

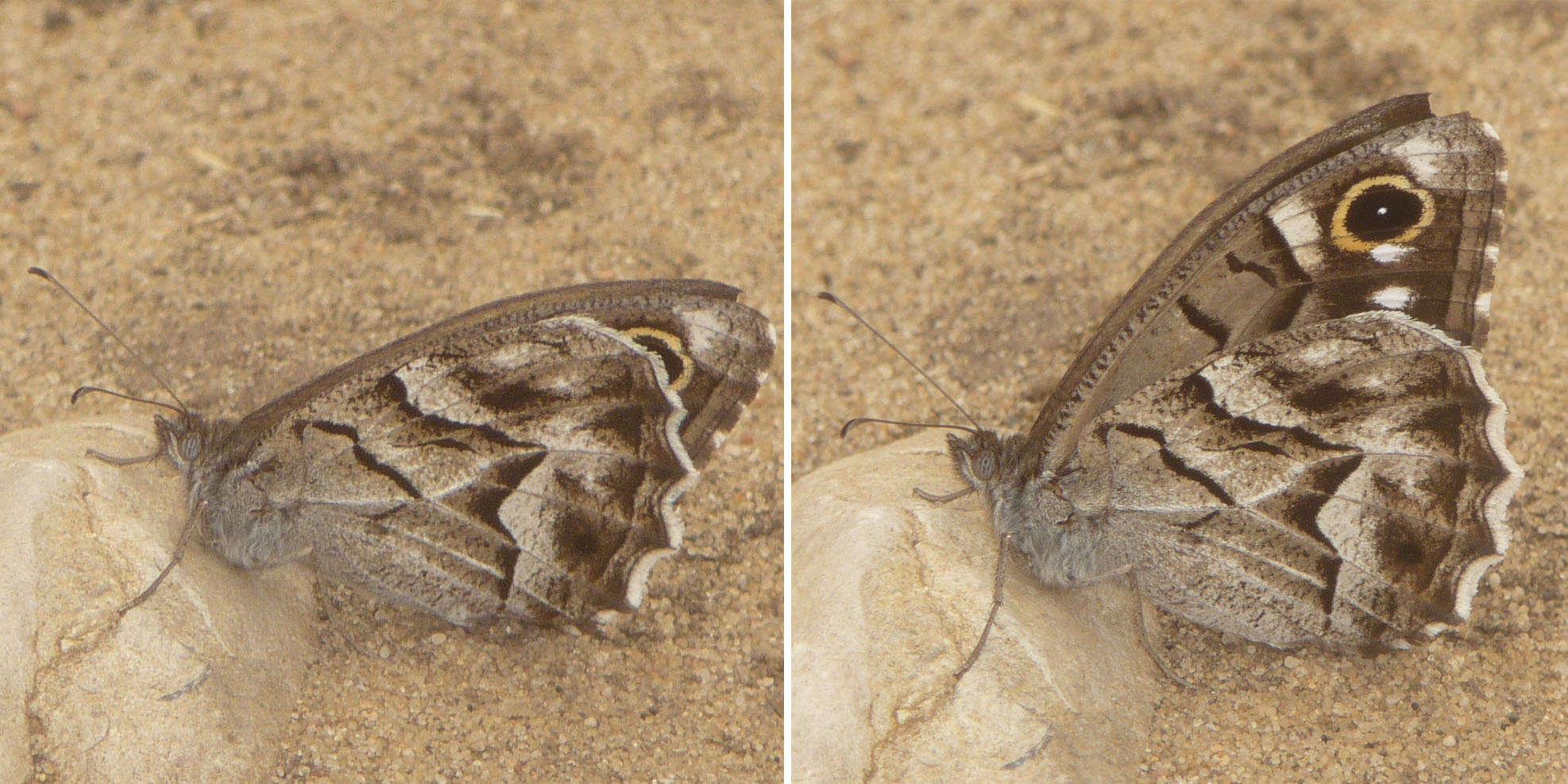

Supplement: S1 Fig — This behavior appears to be especially common in DS forms of B. anynana in the field (M. de Jong, pers. comm.). (JPG) [file pgen.1005529.s001.jpg]

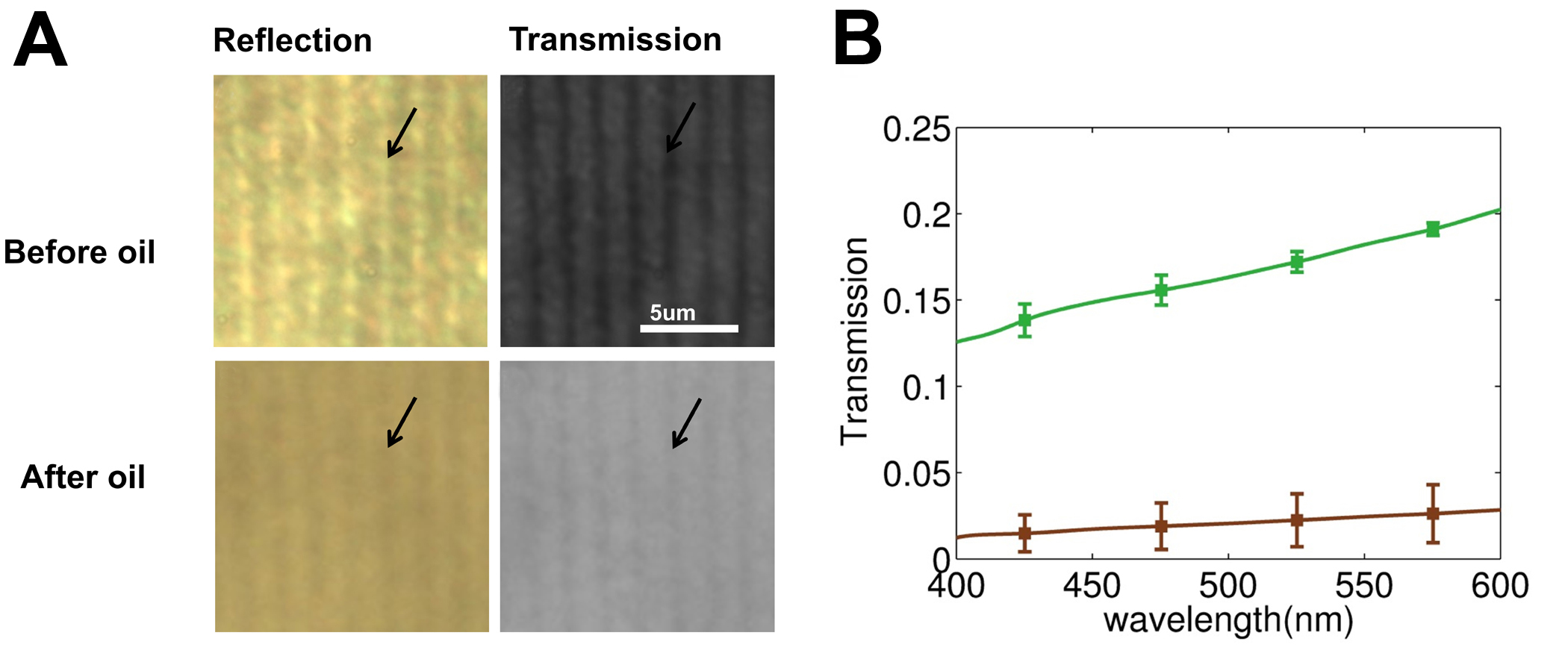

Supplement: S2 Fig — A) Microscopy images of single white scales under epi-illumination and transmitted illumination before and after silicone oil application. Black arrow across all images denotes a ridge where light is mostly reflected. B) Transmission measurements of WS (green line) and DS (brown line) white scales of HWCu1 eyespot after application of silicone oil. Error bars represent the standard error of the mean. (JPG) [file pgen.1005529.s002.jpg]
