# Supplementary material for: Differential Expression of Ecdysone Receptor Leads to Variation in Phenotypic Plasticity across Serial Homologs
Source: PLoS Genet. 2015 Sep 25;11(9):e1005529. doi: 10.1371/journal.pgen.1005529 (PMC4583414; doi:10.1371/journal.pgen.1005529)
Supplement: S1 File — (DOCX) [file pgen.1005529.s003.docx]

**Supplemental File 1:**

**Pigmentation is localized in scale ridges**

To determine where pigmentation is localized in the scales at the center of an eyespot, we isolated individual white/yellow scales (from HW Cu1 eyespots) and observed changes in morphology before and after applying silicone oil (SFig. 2). First, we observed bright, vertical lines spaced 1.4um apart where light was strongly reflected (SFig. 2A-top left). In the corresponding transmission image (SFig. 2A-top right), we observed dark lines that coincided with the bright lines of SFig. 2A-top left, indicating lower light transmission in these areas. After applying silicone oil, scale reflectance decreased, and the scales became transparent (SFig. 2A-bottom left). Surprisingly, the bright lines that were observed before oil immersion became darker, and the reflection image became similar to the transmission image (SFig. 2A-bottom right). These results indicate that scale pigmentation is primarily localized in the scale ridges, and these ridges are also the sites from where light is strongly scattered. To confirm that DS and WS scales have different amounts of pigment deposition, we measured the transmission of the white/yellow centers soaked with oil. After oil immersion, light reflection is suppressed, and scale color depends only on the different levels of pigmentation. We observed a higher transmission for WS scales compared to DS scales, confirming that WS scales absorb less light and have lower levels of pigmentation than DS scales (SFig 2B).
